# Mechanism of Rock Mass Detachment Using Undercutting Anchors: A Numerical Finite Element Method (FEM) Analysis

**DOI:** 10.3390/ma17184468

**Published:** 2024-09-11

**Authors:** Andrzej Wójcik, Kamil Jonak, Robert Karpiński, Józef Jonak, Marek Kalita, Dariusz Prostański

**Affiliations:** 1 Department of Machine Design and Mechatronics, Faculty of Mechanical Engineering, Lublin University of Technology, Nadbystrzycka 36, 20-618 Lublin, Poland; 2 Department of Technical Informatics, Lublin University of Technology, 20-618 Lublin, Poland; 3 Department of Clinical Neuropsychiatry, Medical University of Lublin, 20-059 Lublin, Poland; 4 KOMAG Institute of Mining Technology, Pszczyńska 37, 44-100 Gliwice, Poland

**Keywords:** sandstone, rock mechanics, rock cone failure, FEM, anchor

## Abstract

Undercutting anchors are structural elements used in construction and geotechnics to stabilize both structures and soils. Their main applications include stabilizing slopes and embankments, reinforcing foundations, and providing support during tunnel construction and other underground works. The authors propose the use of these anchors in rock mass detachment technology. This article presents a comprehensive analysis of the mechanism behind rock mass detachment using an undercutting anchor. Particular attention is given to the influence of parameters such as the fracture energy of the medium and the coefficient of friction between the medium and the anchor head on the detachment process of rock elements during anchor expansion in the drilled hole. Numerical FEM analysis was employed to model the effect of changes in the shape and size of failure cones under varying simulation conditions. The discussed problem is crucial for evaluating the effectiveness of this anchor design under non-standard conditions, particularly in the unconventional destruction of rock media.

## 1. Introduction

Undercutting anchors are used in the construction industry and geotechnical engineering to stabilize excavation walls and slopes and to protect against slope failures [1,2,3]. The rapid development of Offshore Renewable Energy (ORE) has driven advancements in innovative anchoring systems, including the use of undercutting anchors for securing components in such installations [4,5,6,7]. These anchors are also used for reinforcing existing structures such as buildings, bridges, and dams, as well as in the mining industry, particularly in tunneling operations for stabilizing excavations [8,9,10]. These anchors transfer loads to more stable layers of the medium, ensuring both safety and efficiency in engineering projects [11].

The authors suggest using undercutting anchors for cutting rocks, a method primarily applied in the mining and construction industries to enable controlled rock removal. In this method, anchors are installed and tensioned in the rock, generating cracks and facilitating the fracture of rock elements. This process minimizes the risk of uncontrolled fractures, ensuring safety, especially in areas near buildings or critical infrastructure.

The authors propose a modified design of undercutting anchors for use in the non-standard extraction of rock lumps. This technology is used in specific engineering situations, such as when rock or concrete structures must be removed and environmental or technical limitations prevent the use of other methods, such as explosives or drilling machines. Due to the complexity and cost of experimental tests, the analyses were conducted using the finite element method (FEM). This method reduces both the cost and scope of experimental tests by enabling virtual simulations, which diminish the need for expensive physical tests [12,13,14,15].

FEM enables project optimization prior to implementation, increases test accuracy, and accelerates the entire research process [16,17,18,19]. These simulations are safer in fields with high risk to human life, eliminating the need for risky and costly physical experiments. The finite element method (FEM) significantly improves the analysis and optimization of rock extraction processes using undercutting anchors. FEM allows precise modeling of rock behavior under anchor impact, optimization of anchor placement, and analysis of their effect on medium stability. These simulations reduce the need for physical tests, lowering both costs and time required for experiments. Additionally, FEM enhances operational safety by identifying potential risks, which is crucial for the protection of both workers and infrastructure.

Typical anchors are generally used in the assembly of technical infrastructure in concrete structures. Currently, there are various design and technological solutions in the field of this assembly technology [20,21]. Anchors can be mounted mechanically, chemically, or during the pouring of concrete [22]. Each of these anchor types has a different design and installation method [23,24]. Since their introduction, efforts have been made to develop computational procedures to determine their load capacity, as well as to understand the factors affecting this parameter, including the mechanical properties of concrete, the design parameters of the anchors, and the installation methods [25].

During the initial trials of developing the proposed cutting technology, the authors relied on the existing body of knowledge, particularly regarding the mechanical impact of anchors on the medium in which they are mounted, with a focus on undercutting anchors [26,27,28]. These anchors are used in the installation of steel elements and infrastructure, such as telecommunication systems, in typical concrete structures [29] as well as in composite structures [30,31]. Empirical studies have established that, for these solutions [32,33,34,35], a commonly used formula for estimating anchor load capacity is:

(1) $$N_{u,m}=k_{1}f_{c}^{0.5}h_{ef}^{1.5}$$

where

*N_u,m_*—mean pullout load (load capacity of anchors) [N];
*k*_1_ [N^0.5^/mm^0.5^]—calibration factor, accounting for, e.g., units in the model, anchor type, base material, embedment depth, etc.;
*f_c_*—compressive cylinder strength of concrete [N/mm^2^];
*h_ef_*—effective embedment depth [mm].

As it results from Equation (1), load capacity of anchors depends mainly on effective embedment depth *h_ef_* and rock compressive strength *f_c_*.

To increase an accuracy of estimating the load capacity of anchors, calculations are often supported by artificial intelligence (e.g., neuronal networks) [36].

In turn, based on linear-elastic mechanics of cracking, it was established [25] that the anchor load capacity can be determined from the relationship

(2) $$F_{EG_{f}}=2.1{\left(EG_{F}\right)}^{0.5}h_{ef}^{1.5}$$

where

$G_{F}$—the fracture energy of concrete;
*E*—the modulus of elasticity of concrete

The load capacity (anchor pullout strength) *F_EGf_* [N] is a function of the calibration factor a_1_, the anchoring depth *h_ef_* [mm] in the exponent 1.5, the modulus of elasticity of concrete *E* and the fracture energy of concrete *G_F_*. The critical calibration factor a_1_ = 2.1[N^0.5^/mm^0.5^] links the anchor load capacity (peak load) with the length of the propagating crack at loss of stability, compared to its extrapolated total crack length to the concrete surface, while a—actual crack size measured along the crack path, *l_B_* is the projected crack path (at an angle of 37.5° to the horizontal direction) in [mm].

**Figure 1:**
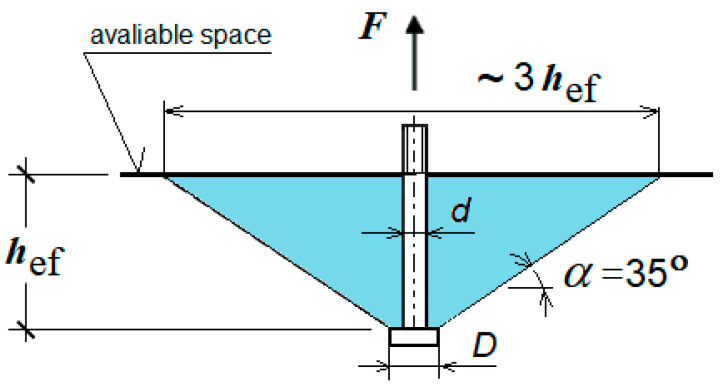
Approximated concrete breakout cone for tension: *h_ef_*—effective embedment depth, *α*—breakout prism angle, *d*—anchor shaft diameter, *D*—anchor head diameter, *F*—anchor load force.

Research [4,6] using the geotechnical particle finite element method has shown that for shallow anchoring depths, a classical failure cone with an angle of α ≈ 27.5° forms. For greater anchoring depths, rock plasticization primarily occurs around the largest diameter of the anchor head, causing the failure cone angle to decrease. In the tests conducted thus far, it has been shown [34] that the traditionally used model of damage zone of concrete, in a form of a so-called breakout cone, (Figure 1) is too big of a simplification in relation to rocks, in particular sandstone.

**Figure 2:**
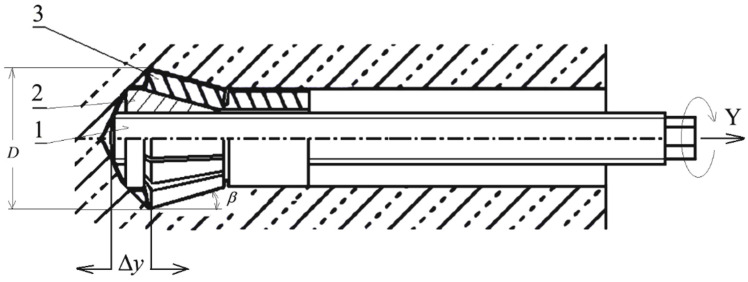
A modified design of breakout anchor: 1—driving screw, 2—conical nut, 3—segment spring sleeve, *β*—angle of conical head, Δy—relative displacement of the conical head and of the anchor driving screw end, *D*—anchor head diameter, Y—direction of the coordinate system in accordance with the anchor axis.

With constant anchor stiffness, an increase in the anchor head angle *β* (Figure 2) results in a higher load-bearing capacity (pullout force). Additionally, it was found that an increase in the rock’s Young’s modulus leads to a nonlinear increase in the anchor’s pullout force.

It has been verified empirically [34,37] that the procedures used up until the present time are suitable for the estimation of anchors’ load capacities (and therefore also the forces pulling out anchors) mounted in concrete, as described in [38,39], and to determine the conditions of pulling out anchors mounted in rock media (mines of sandstone: Brenna, Braciszów, and Guido, Zalas mine of porphyry). It has been stated by the authors that in the case of estimating the load capacity of anchors (as well as pullout forces), it is indispensable to introduce significant corrections of the exponent coefficients in the existing computational formulae.

The majority of analyses concerning the reaction of anchors to concrete are realized numerically, mainly with the use of FEM [40,41,42], BEM (boundary element method) [43], or a hybrid finite–discrete element method (FDEM) [44,45,46,47,48]. 

In our numerical analyses using FEM [49,50], it was determined (and field tests confirmed [34]) that the damage zone of the rock medium generated during an anchor pullout differs significantly in both shape and range (measured on the rock’s free surface) from the commonly used breakout cone model developed for concrete (3 *h_ef_*, Figure 1). The sizes of the α separation angle are significantly smaller than those observed for concrete (about half the size). It should be noted that the cone-shaped fracture is sufficient for estimating the maximum force required to pull out the anchor, as it was developed for this purpose. However, for estimating the practical development of cracks, which affects the entire crack trajectory and the real scope or volume of separation, such an approach is overly simplistic. In these cases, one of the aforementioned methods (FEM or BEM) is more appropriate. 

From a series of tests concerning anchors mounted in concrete, it can be concluded [51,52,53] that, apart from the effective embedment depth and the concrete compressive strength, the anchor geometrical parameters—such as the ratio of the anchor screw diameter d to the head diameter D (Figure 1), or the ratio of these dimensions to the effective embedment depth—are essential for the progression of damage in the medium structure. Therefore, for undercutting and extracting anchors, using flat [54], axial and symmetrical [49], and 3D models [55], we conducted extensive tests aimed at establishing the impact of parameters such as the anchor head diameter D [56] and the angle of the head’s conical part β (Figure 2) [57] on the formation of the initial separation angle *α_0_* and the trajectory of crack propagation. Additionally, the influence of the mechanical properties of the rock medium [58], such as Young’s modulus *E*, Poisson’s ratio *v*, the fracture energy of rock materials *G_F_*, and the friction coefficient between the head and the rock *μ*, was taken into consideration.

In the result of the tests conducted thus far, the following has been stated by us:

- When the head friction coefficient on the rock increases, a limitation of the fracture range occurs and an increase in the initial separation angle *α_0_* takes place;
- An increase in the strength of rocks against breaking causes a limitation of the fracture range, an increase in the initial angle of separation, and a change in the trajectory of tracks for a parabolical one, whereas for a small value more complex trajectories of cracks occur;
- An increase in the Poisson ratio causes a reduction in the initial angle of crack propagation and an increase in the range of fractures;
- An increase in the Young’s modulus favors a limitation of the fracture range and an increase in the initial angle of crack propagation;
- An increase in undercutting head diameter, for other fixed parameters, does not cause any essential changes in the conditions of crack propagation in the initial phase of its development;
- An increase in the undercutting head angle limits the scope of separation and favors an increase in the initial crack propagation angle.

Treating the undercutting-and-extracting anchor as a tool of multiple application [57,59], we analyzed the impact of its potential blunting during exploitation, leading to a change in the undercutting head angle *β* (Figure 2) as well as of its diameter *D* on shaping the crack trajectory during the breaking of the rock in the result of the anchor action. As the result of its blunting, the angle of the undercutting head, together with a simultaneous decrease in the head diameter, is reduced. This leads to the occurrence of similar effects as described above. 

Based on the current state of knowledge concerning the interactions between anchor assemblies and concrete [60,61], we also analyzed the interaction of anchor assembly cones mounted and pulled out in rock media [10,14,62]. We found that, in the case of rock, these interactions occur over a larger area, which is related to the smaller fracture cone angles in rock compared to concrete. Determining the optimal arrangement of anchor holes is essential to minimize the required pulling forces and to maximize the fractured volumes generated by potentially used sets of anchors.

Based on experience gained from the use of pulled-out undercutting anchors, and due to the difficulties encountered during operation—stemming from the overall dimensions and the weight of the devices required for pulling out anchors [37,63]—the authors proposed a completely different method of transferring the load from the anchor to the rock, used in undercutting-and-extracting anchors [64]. The first field tests, along with numerical analyses [56], indicated that this was a promising development direction for the proposed loosening technology. However, new elements requiring clarification have emerged, such as discrepancies in the location of crack initiation points and their propagation when comparing the impact of the new anchor design on the rock medium to the behavior observed in the case of pulled-out anchors. In the case of pulled-out undercutting anchors, crack initiation traditionally begins at the corner of the undercutting head. However, for undercutting-and-extracting anchors, expanded in the area of the hole bottom, tests conducted thus far have shown that crack initiation occurs at the support point of the anchor’s driving screw. Since the location of crack initiation and the initial propagation angle of cracks (α_0_) are crucial for determining the fracture zone [64], there is a need to verify the current understanding of the breaking mechanism, the extent of the damaged zone under specific technological conditions, as well as the geometric parameters of the anchor head and driving screw. This justifies the development of a new design for the undercutting-and-breaking anchor.

## 2. Materials and Methods

In the current analysis, the object of the tests involved a new, simplified design of a breakout anchor, shown in Figure 2. A characteristic feature of this anchor’s operation is the breaking of the rock medium at the bottom of the hole where it is installed. This mechanism of medium damage differs from that of classical undercutting anchors, where the medium fractures as a result of rock lumps breaking off during the anchor pullout. In the solution used thus far, the process of pulling out the anchor has required the generation of significant pulling force with the help of auxiliary devices, which are large and heavy, posing a major limitation, especially during rescue operations in confined spaces, such as damaged mine workings. Therefore, alternative solutions for both anchor design (e.g., Figure 2) and the process of breaking the medium structure have been explored. One of the designs under consideration is presented in [56].

The breakout anchor of the new design consists of a driving screw (1), on which a conical nut (2) and a spring-and-adapter sleeve (3) are mounted. The conical nut, together with the adapter sleeve, is hereafter referred to as the conical head of the anchor. To place the anchor in the rock, it is necessary to first drill a hole with an undercut for the head. Figure 1 shows the anchor in its position after installation, placed in the pre-drilled hole with an undercut for the anchor head. This anchor design can be compared to a uniaxial screw mechanism. Screwing in the driving screw (1), which is supported at the hole bottom, into the conical nut (2), which is expanded between the screw and the previously created conical undercut in the rock, causes a gradual increase in the dimension Δy (Figure 1) between the zones where these elements are supported on the rock. As a result, rock deformations increase, leading to successive cracking until the crack reaches the free surface, ultimately breaking out a rock element. In the studies conducted thus far, particularly in standards, the shape of this separation has been approximated by a so-called cone of damage [22], which is a significant simplification when applied to rocks.

The expansion sleeve (3, Figure 1) of the proposed anchor head design typically consists of six taper-spreading segments. However, unlike the Hilti anchor (e.g., type HDP-A), these segments do not create a taper undercut in the rock. This undercutting, which is necessary to spread the anchor within the hole, must be performed as an additional operation using specialized tools.

At present, the impact of a new anchor design on the mechanism of rock medium fracturing, particularly in terms of understanding how the contact zone localization of anchor elements (driving screw, anchor head) with the rock affects the initiation point of rock cracking and the initial crack trajectory, requires more detailed investigation. This includes how these factors influence the process of medium fracturing and the resulting scope of rock breakage. This aspect is crucial for evaluating the effectiveness of the proposed technology, which aims to extract the maximum amount of rock with minimal labor effort during a single anchor fixation.

Research problem—In the elaboration, special attention was paid to the impact of the friction coefficient of the new anchor head and the rock and the fracture energy of the rock medium on the localization and shaping of the breakout zone of the rock medium at the initial stage of crack development leading to the extraction of rock lumps. The concrete fracture energy is the amount of energy needed to open a single crack area.

The article presents recent research findings and serves as a supplementary analysis to the study [64]. It focuses on the impact of rock fracture energy and the coefficient of friction between the rock and the anchor head within the context of a new version of stripping technology, specifically examining their influence on the configuration and propagation of the fracture trajectory. This, in turn, affects the volume of the extracted ore body, which is crucial for evaluating the efficiency of the proposed technology.

Numerical analysis of the breakout process with the use of the anchor of the discussed design was conducted with the application of the algorithm extended finite method (XFEM) [65,66,67,68,69] in the software ABAQUS (Abaqus 2023, Dassault Systems Simulia Corporation, Velizy Villacoublay, France). An advantage of this algorithm is that the network of finite elements has minimal significance as regards the precision of the obtained simulation results [70].

A type of material and its mechanical parameters were assumed for an analysis: Sandstone: Elastic, isotropic. Elastic modulus—*E* = 14.276 MPa, Poisson’s ratio—*ν* = 0.247, Tensile strength—*f_t_* = 7.74 MPa.
Anchor material: Steel: Elastic, isotropic, elastic modulus—*E* = 210,000 MPa, Poisson’s ratio—*ν* = 0.3.
The friction coefficient of steel head and the rock was assumed as follows: *µ* = (a) 0.2, (b) 0.4, (c) 0.6 (three theoretical cases were analyzed).

The material parameters for the model were selected to closely align with those observed in field studies at aggregate extraction sites. Concurrently, the impact of various mechanical parameters of the examined rocks has been analyzed in prior publications. The results presented here augment and expand upon previous analyses by examining the influence of fracture energy—an aspect not yet explored for the new anchor model. Additionally, the study investigates the effect of the friction coefficient at the rock–anchor head interface on the fracture trajectory, specifically for the newly designed anchor solution.

Field observations of the detachment process revealed that, in the case of sandstones, detachment using an undercutting anchor typically results in brittle fracture, characterized by a discrete surface (trajectory) of material failure. Consequently, the decision was made to employ the brittle material model available in ABAQUS for numerical simulations. In this context, the critical mechanical parameters are the tensile strength *f_t_* (as the crack initiation criterion) and fracture energy *G_F_* (as the failure propagation criterion). Additionally, due to the interaction between the anchor head and the rock—which involves friction and the sliding of the rock on the conical surface of the anchor head (a contact issue with friction)—the Coulomb friction coefficient *μ* is of significant importance. These factors, in our assessment, substantiate the inclusion of these parameters in the numerical simulation of the interaction process between the anchor head and the rock.

One of the primary challenges in applying the finite element method (FEM) for failure analysis in rock media is the lack of sufficient experimental data required to accurately simulate material properties [71,72,73,74]. Additionally, in damage models that rely on fracture energy, the precise selection of energy values is crucial for accurately modeling the detachment process. Given the significant variability in the physical and mechanical properties of actual rock media, determining fracture energy values is often challenging and requires complex testing procedures. Moreover, such tests do not always yield reliable results, particularly for highly brittle rocks, which tend to disintegrate rapidly upon fracture, making it difficult to accurately estimate fracture energy, even with high-speed imaging. A similar challenge arises with the coefficient of friction between the anchor head and the rock surface, which is highly dependent on factors such as the moisture content and grain size of the rock. In practice, these parameters can vary significantly within a single deposit. Therefore, this paper examines the influence of fracture energy and the Coulomb friction coefficient on fracture trajectory and rock element formation under various simulation conditions, assuming theoretically plausible values for these parameters. The objective is to identify potential trends in crack propagation under conditions analogous to those observed in field tests.

Geometry of anchor and parameters of the pulling-out process
Depth of anchorage *h_ef_* = 100 mm;
Angle of head *β* = 15°;
The hypothetical fracture cone angle was initially assumed as *α* = 22.5°.

For the analysis of the rock medium cracking, due to the action of the breakout anchor, the typical procedures, determining the conditions of the initiation and development of damage, implemented in the software FEM ABAQUS were used, i.e.,

Damage initiation in rock material: Maximal principal stress,
Damage evolution type: Energy, softening linear.
Damage for traction—separation laws: Maximal principal stress damage;
Fracture energy *G_F_* = (a) 0.17, (b) 0.355, (c) 0.7 (N/mm)—three theoretical cases were analyzed (in a particular case, linear issues, the fracture energy *G_f_* is coincident with the critical strain energy release rate *G_Ic_* = *K_Ic_*^2^/E [75,76,77,78], where *K_Ic_*—critical stress intensity factors, *E*—elastic modulus). Damage stabilization: Cohesive.

The material data used in the simulations were chosen to correspond to the actual rock parameters of the rock mass where the field tests were conducted. Additionally, they were derived from the characteristics of the material model used in ABAQUS (the brittle material model) and the criteria adopted for the initiation and propagation of failure in such materials.

Geometry of the model

Rock medium

**Figure 3:**
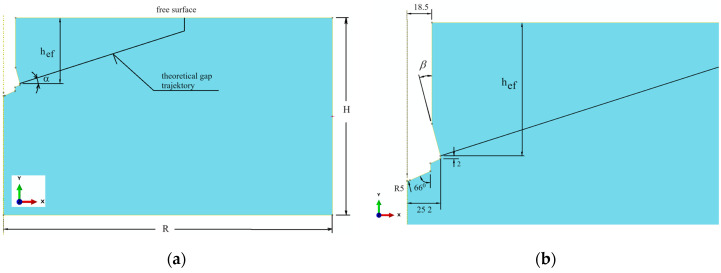
(**a**) Geometric parameters of the rock medium model, (**b**) characteristic dimensions of a hole with an undercut for the anchor, where *h_ef_*—effective anchorage depth, *α*—theoretical angle of the “damage cone”, *H*—height of the medium model, *R*—radius for the axially symmetric model, *β*—angle of conical head (unit: mm).

A flat geometric model was used. Due to the model symmetry, the axially symmetric model of the following dimensions was applied: length *R* = 500 mm, height *H* = 300 mm (Figure 3). Therefore, in the rock model the model with a half-size hole for the anchor of diameter *ϕ* = 37 mm (for M20 anchor), Figure 3b, was introduced. It was also assumed that an undercut was made earlier for the anchor head, required for its assembly in the rock material.

A part of the anchor installed in the hole made in the rock medium and prepared for its expansion to damage the rock by breaking it at the bottom of the hole is presented in Figure 3a. In the interaction zone between the anchor elements and the rock (Figure 3b, marked in yellow), the “surface-to-surface” contact with friction, using the “penalty contact” method available in the ABAQUS software, was applied.

**Figure 4:**
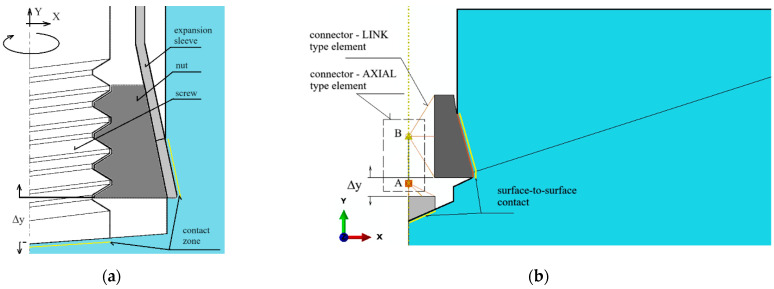
A part of anchor installed in the hole prepared for its extension to damage the rock due to breaking out—(**a**) method of modeling the relative movement of the driving screw and the anchor conical head during a screw turn, (**b**) Δy—distance between the A and B nodes of the linear connector.

According to the principle of a screw mechanism, turning the screw around its axis causes a linear movement of the nut (anchor head) along the same axis (Figure 4a). As a result, the dimension Δy, which determines the relative position of the screw tip and the anchor head, increases. In the initial phase of movement, the rock in the contact zone with the anchor elements, i.e., beneath the screw tip and in the conical part of the anchor head, undergoes elastic deformation. The stresses in the rock gradually increase. Once the principal stresses reach a critical value, equal to the rock’s tensile strength *f_t_*, a crack is initiated. The increasing distance Δy between the anchor elements leads to the progressive development of the crack during the rock’s fracturing, until a rock element (the so-called cone of damage) is broken out.

For the purposes of numerical analysis of the rock separation process caused by the action of the anchor head, the mechanical model shown in Figure 4b was adopted. To simulate the kinematic forcing of the anchor head relative to the end of the driving screw, which is supported at the hole bottom, reference points connected to the anchor elements, as depicted in Figure 4b, were used in the simulation. Thus, to model the relative movement between the screw end and the conical head (Figure 4b), the “Connector—AXIAL type” and “Connector—LINK type” finite elements available in the ABAQUS software were utilized. The “AXIAL” type connector establishes a link between the model’s nodes (A and B, Figure 4b), which acts along the line connecting these nodes, allowing only relative movement along the axis between these elements. In ABAQUS/Standard, a node or a group of nodes can be constrained to a reference node. Similar to multi-point constraints, the kinematic coupling constraint allows for the specification of constrained degrees of freedom on a node-by-node basis.

For reference points kinematically coupled (U1 = 0, U2 = 0, UR3 = 0) with the anchor parts, kinematic forcing was applied in the form of a displacement ΔU2max = 10 mm (Δy—Figure 4b). This allows movement of the anchor head relative to the screw end (hole bottom) only along the *OY* axis of the defined coordinate system.

As shown in Figure 4b, the reference nodes of the conical head model were connected to node B of the “AXIAL connector” using a multi-point kinematic coupling of the “LINK connector” type. This allows for the potential movement of the head together with node B along the *Y*-axis (the axis of the anchor). Similarly, the reference points of the driving screw end were connected to node A of the “AXIAL connector” using a multi-point kinematic coupling of the same “LINK connector” type. This allows the screw end to move together with node A along the *Y*-axis (the screw axis). As a result, a relative movement between the anchor components is possible along the screw axis (the hole axis in the rock), with the increasing distance Δy between points A and B of the connector (representing an increase in the distance between the anchor components). This movement can also result in deformations of the rock medium due to the action of both the screw end and the conical head.

In conclusion, the reference points were kinematically coupled (degrees of freedom U1, U2, UR3) with parts of the anchor and the screw. Kinematic forcing in the form of increasing displacement along the connector axis was applied, with a maximum value of ΔU = 10 mm. The elongation step, starting at an initial value of 0.001 mm, increased to a maximum of 0.01 mm, provided the convergence of the calculations was maintained.

Boundary conditions of the model

**Figure 5:**
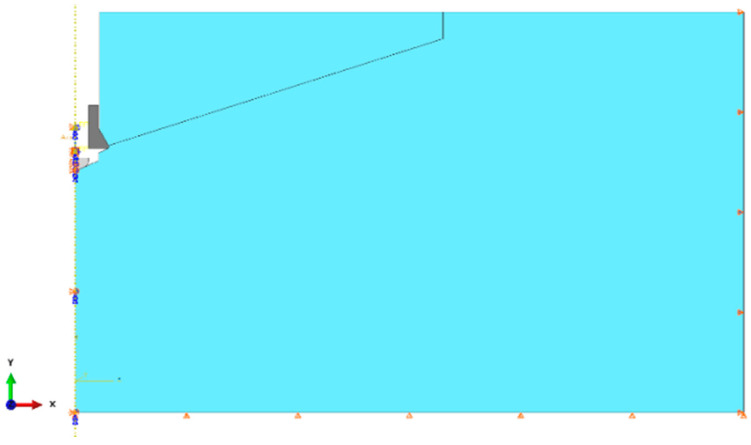
Restraints of boundary elements of the model.

The restraints/boundary conditions are shown in Figure 5. The node in the vertical axis of the model at the bottom has been stripped of all degrees of freedom. Restraints include the following: nodes in the base of the model—U2 = 0, nodes on the right edge—U1 = 0. In the model of rock and reference points connected with the anchor and screw, there is symmetry in relation to the *Y* axis; therefore, U1 = U3 = UR2 = 0 (Figure 4).

As it has already been mentioned in the zone of interactions between anchor elements and the rock (tangential contact), the procedure “surface-to-surface contact” available in the software ABAQUS was used. Contact Property Options: Tangential Behavior. Friction Formulation: Penalty. Friction Coefficient: *µ* was applied.

Finite element mesh of the rock medium model

**Figure 6:**
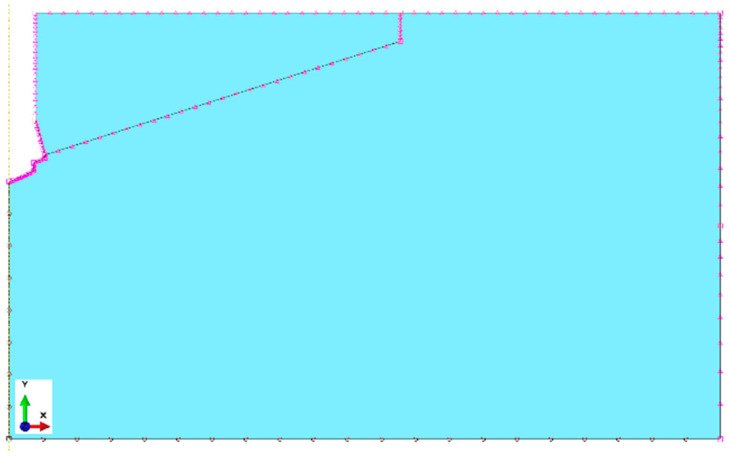
Distribution of nodes in the finite element mesh along the characteristic edges of the rock model.

Quadrangular elements in the free arrangement, elements of the type CAX4R, four-mode, linear, and with reduced integration were used. For a construction of the finite element mesh, the elements of the maximal linear dimension 25 mm with local concentrations on the edges up to 1 mm were applied. The obtained distribution of elements/nodes of the finite element mesh of the rock model along the characteristic edges of the model is presented in Figure 6. In the essential areas of the model, a differentiated size of finite elements was used, taking advantage of an automatic generator of the mesh, imposing an input size of the element in the determined points of the model. Therefore, the total size of elements (side length) was accepted as 25 mm, whereas in the contact area with the anchor conical head 2 mm was accepted; in the area of the screw end contact with the hole bottom surface, a value from 0.4 to 1 mm was applied; and along the line of the forecasted crack, 5 mm was accepted. For the edge in the lump top part, the sizes of elements were differentiated, i.e., from 3 to 10 mm (in relation to the rock model zone).

As can be seen in Figure 6, in the zone of the potential crack initiation (zone of screw end contact and the undercutting head with the rock), a high concentration of nodes/elements was obtained.

The research [70,79,80,81,82,83,84] shows that the XFEM algorithm used in the presented numerical analysis is insensitive to the size of the finite elements of the model mesh.

The publication [64] presents an analysis of the model’s response sensitivity to the element size in the finite element mesh.

**Figure 7:**
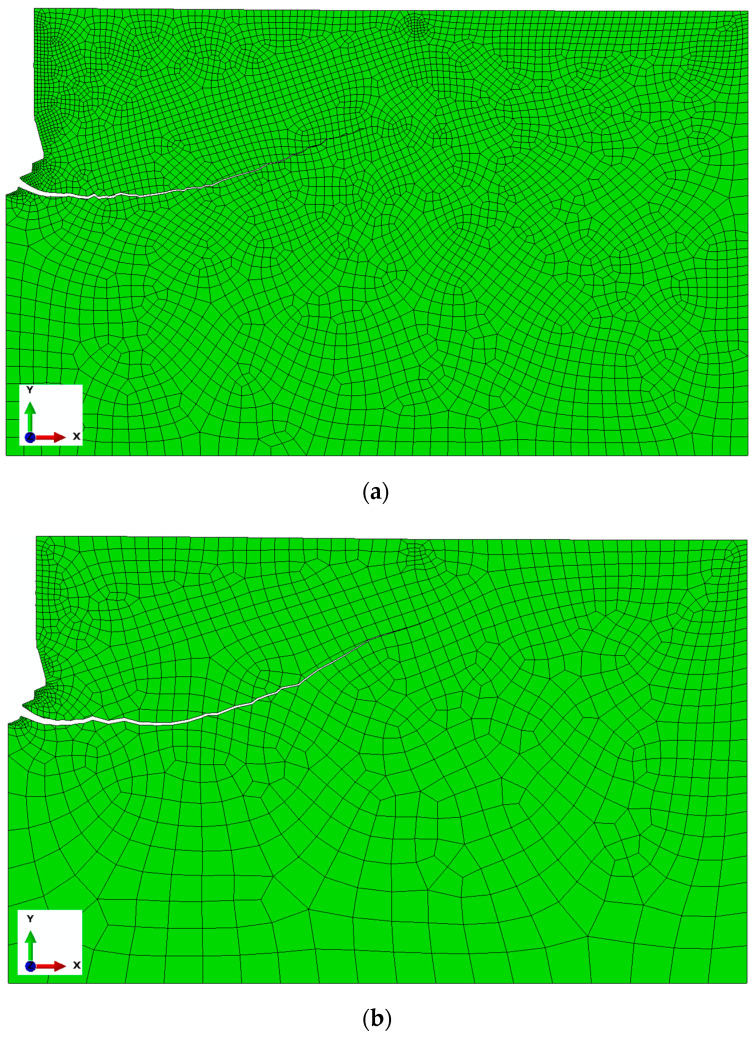

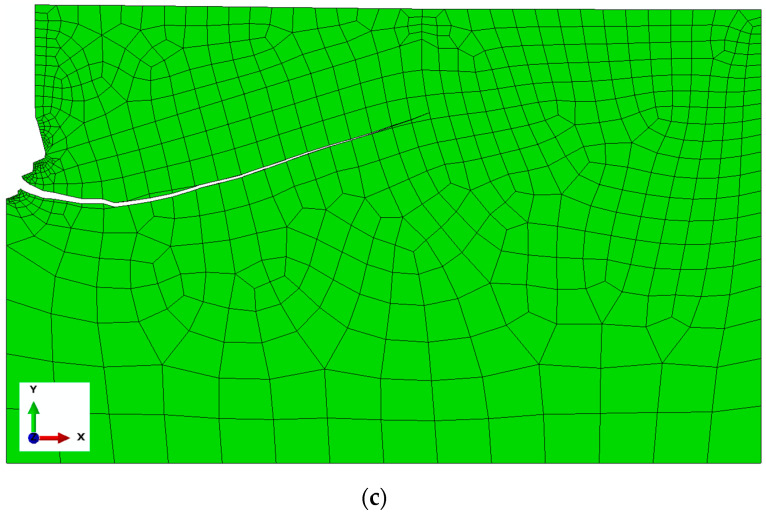
Sensitivity of the rock model to the size of the finite elements, for proportions of the nominal dimensions of the model equal to (**a**) 0.5, (**b**) 0.75, (**c**) 1.5.

These studies confirmed the validity of previous reports regarding the pullout anchor model. For the issue at hand, specifically the modified stripping process and the new anchor design, verification analyses were conducted, the results of which are presented in Figure 7. In the anchor model, the finite element mesh remained unchanged. In the rock model, the mesh density at the edges of the hole also remained unchanged. However, the mesh density in the rock model was altered, particularly the global element size at the edges and along the predicted fracture line. Three models were considered with mesh densities proportional to the nominal value (used in the calculations), which were 0.5, 0.75, and 1.5.

The anchor head model with angle *β* = 15°, friction coefficient *μ* = 0.2, and fracture energy *G_F_* = 0.17 N/mm was used for calculations.

**Figure 8:**
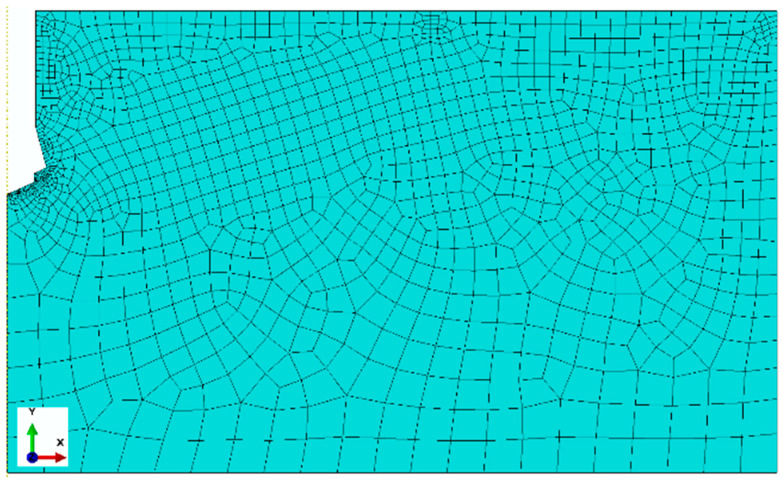
Mesh of finite elements of the rock model.

As shown in Figure 7, the size of the mesh elements affects the smoothness of the po-surface/trajectory of the slot. It also significantly affects the number of iterations as well as the required computation time. The optimal mesh (base mesh), constructed as a result of the authors’ previous experience and the above experiment, selected for further analysis is shown in Figure 8. The mesh generated this time contains 1456 nodes and 1365 elements of the CAX4R type.

## 3. Results

### 3.1. FEM Model Results

**Figure 9:**
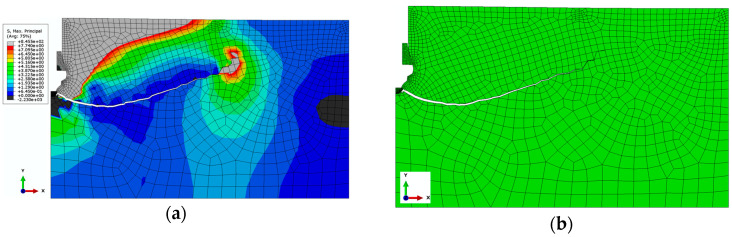
(**a**) Distribution of stress *σ_max_* in the vicinity of the anchor head and generated slot, (**b**) mesh of finite elements of the rock model together with the generated slot (in the increased scale of deformation).

Exemplary results of the conducted analysis are presented in Figure 9. This case concerns an anchor of the head angle *β* = 15°, for a friction coefficient of the anchor and the rock of *µ* = 0.2.

To illustrate the generated slot in a better way, deformations in the increased scale (x5) which presents a bigger spacing of the slot are shown in Figure 9.

**Figure 10:**
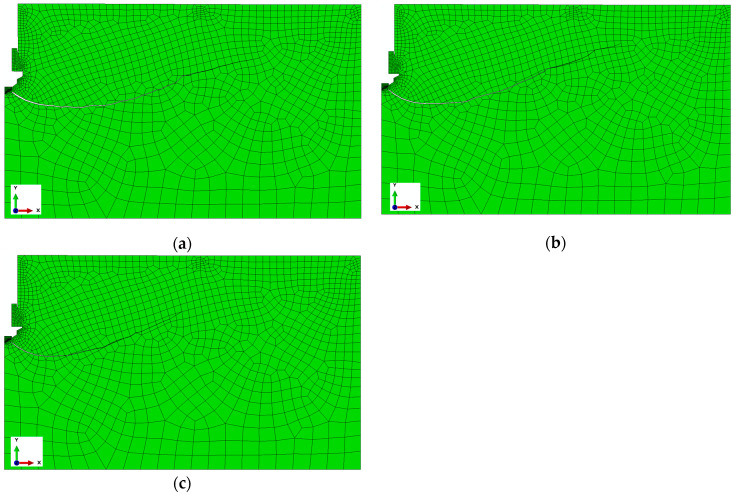
Impact of friction coefficient of the head and the rock *µ* on the slot propagation: (**a**) *µ =* 0.2, (**b**) *µ =* 0.4, (**c**) *µ =* 0.6.

Due to the undertaken trial determining the impact of the friction coefficient of the anchor’s new version and the rock in Figure 10, the slots obtained, respectively, for the friction coefficients *µ* = 0.2, 0.4, and 0.6 are presented. These are hypothetical values, serving for the determination of the tendencies of changes in the course of the generated slot, are shown in relation to a change in this coefficient (the determination of a real friction coefficient is quite a difficult problem, most often its values in simulations are established on the principle of approximation, in relation to other friction processes in technology) [85]. In one study [86], it was demonstrated that for sandstones, the average values of the friction coefficient were approximately 0.589. To present this issue in a better way, the slot is presented on a model; however, it is presented in the option without an illustration of the stress distribution (one of options of the ABAQUS software).

The obtained tendency of changes is shown in Figure 10, where in one image different courses of slots are imposed (in the option without showing the mesh) for the values of the friction coefficient under consideration.

**Figure 11:**
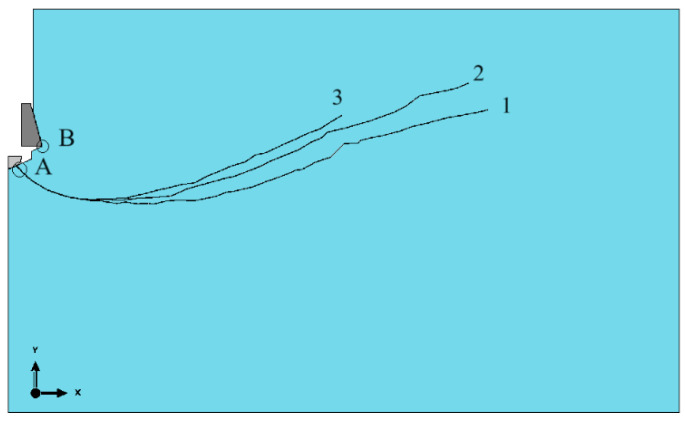
Impact of friction coefficient of the head and the rock *µ*—cumulative presentation: (1) *µ =* 0.2, (2) *µ =* 0.4, (3) *µ =* 0.6. A—initiation point of slots in the contact zone of the anchor driving screw with the rock, B—initiation point observed during a simulation of breaking out a traditional anchor (at conical base of the head of anchors).

From Figure 11, it can be concluded uniquely that an increasing value of the friction coefficient *µ* of the head and the rock medium causes a limitation of the slot range on the rock’s free surface (in favor of limiting the volume of the potentially broken out rock lump—so-called cone of damage). The obtained waveforms are consistent with those observed in [64].

Small coefficients of friction are in favor of shaping further ranges and the creation of bigger rock lumps broken out in the process.

**Figure 12:**
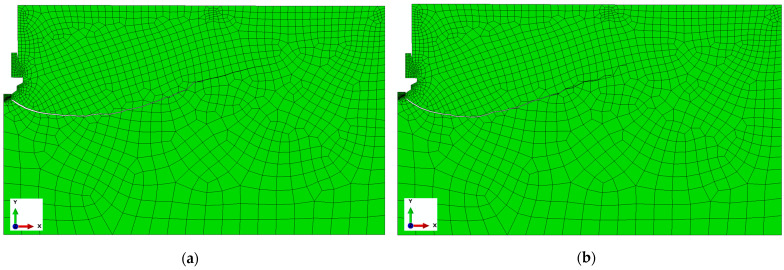

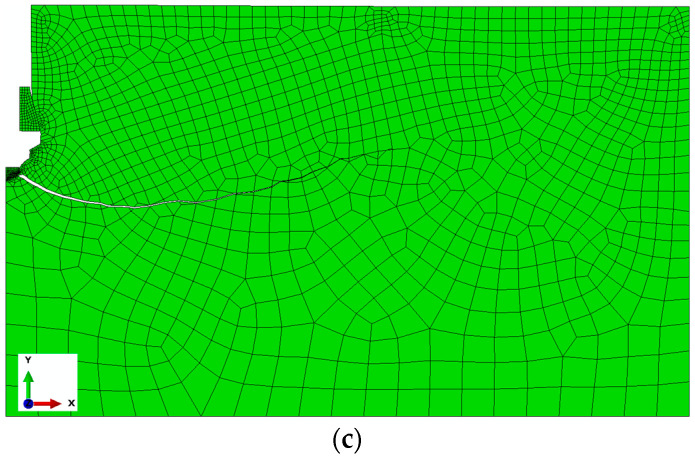
Impact of the fracture energy *G_F_*. on the trajectory of slots for (**a**) *G_F_* = 0.17, (**b**) *G_F_* = 0.35, (**c**) *G_F_* = 0.7 (N/mm).

Bearing in mind another aspect of the conducted analysis, i.e., the impact of the energy rate of rock medium cracking on the course/range of slots and shaping of the volume of potentially broken out rock lumps, in Figure 12 the courses of slots for different values of the fracture energy *G_F_* are presented in relation to other identical simulation conditions, obtained as a result of simulation. Similar to above, the slot is presented on the background of the model finite element mesh.

**Figure 13:**
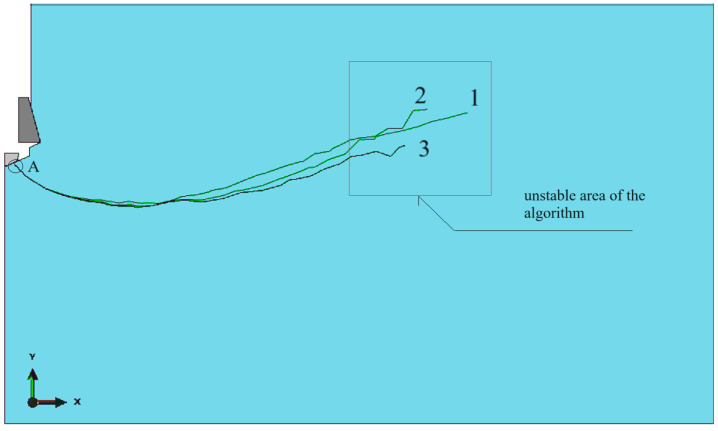
Impact of fracture energy *G_F_* on the course of the slot trajectory—a cumulative presentation for (1) *G_F_* = 0.17, (2) *G_F_* = 0.35, (3) *G_F_* = 0.7 (N/mm).

In turn, in Figure 13, for a better possibility of comparison, a cumulative presentation of the obtained courses of slots for the accepted values of the fracture energy *G_F_* accepted in the simulation is given. These values, for comparative purposes, were accepted mainly as hypothetical; however, they are contained in the area of values characteristic for the rocks under testing [87].

Similar to the case of simulating the impact of the friction coefficient between the anchor and the rock, the initiation points of the cracks were also observed at the end of the breakout screw (point A, Figure 12). Within the stable operation of the crack propagation algorithm, as implemented in ABAQUS software, it was found that an increase in fracture energy promotes the deeper penetration of cracks in the initial phase of their development (e.g., Curve 3). However, in the later phase of crack development (the area marked with a rectangle, Figure 12), the algorithm struggles to uniquely determine the propagation direction at the crack tip, and thus the path of the cracks is not uniquely defined. This issue is described in detail in [88].

During earlier field tests [37,50,54], it was noticed that the course of trajectory of cracking, depends, to a large extent, on the strength parameters of the rock and the head geometry [49,57]. For porphyry and sandstone of large compressive and breaking strength, the slot is generated at the head base and it quickly proceeds to the rock’s free surface [58], along the near-parabola curve.

Comparing the results presented above with those obtained during the initial tests with the undercutting-breakout anchor, a strong convergence can be observed in the courses and initiation points of the generated slots during the breakout of rock elements. This, to some extent, confirms the correctness of the calculations. However, when these results are compared with earlier findings from numerical simulations and field tests, differences arise in the localization of the initiation points of the slots (i.e., the end of the driving screw for breakout anchors through a rock fracture or the corner of the undercutting head in the case of breakout anchors). These differences require further investigation. The key question is whether this discrepancy is due to a certain inaccuracy in the algorithm’s operation in the ABAQUS software (as described in the publication [88]), or if it stems from a more complex issue related to changes in the cross-section of the broken-out element and the distribution of loads generated in the rock during the formation of the slot.

The aspect of the influence of fracture energy in light of the above results is not clear and requires further research. Hence, a comprehensive study of the influence of the compressive strength of the rock medium and its grain size on the formation of fracture energy as well as the course of the failure trajectory is planned. In this study, the FE model for an anchor bolt in uncracked reinforced concrete was simulated at different values of rock fracture energy (i.e., *G_F_* = 0.04, 0.07, 0.11, and 0.15 [N/mm]). It was found that the peak load on the anchor increases with increasing rock fracture energy. Also, the displacement of anchors at peak loads increases significantly with the increase in concrete fracture energy, which results in the more plastic behavior of the anchors. Based on the comparisons, the best agreement with experimental results was obtained for a concrete fracture energy equal to 0.07 [N/mm]. In turn, according to [89], the formation of the angle of the failure cone and therefore the extent of the failure zone of the medium is extremely significantly influenced by the speed of inflicted deformations in the rock (the rate of anchor pullout).

In the case of sandstone-type rocks, it is necessary to conduct more detailed studies, i.e., numerical in this context.

### 3.2. Empirical Validation of the Modeling Results

Initial tests of applying anchors of new design for breaking out rock lumps were conducted during the realization of the TANGO-IV-A/0058/2019 project.

**Figure 14:**
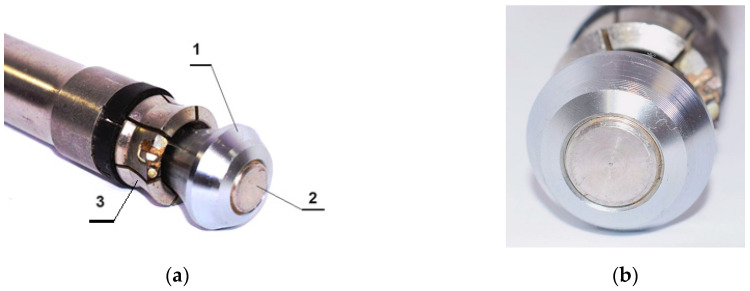
HDP-A undercutting anchor modified and used in the first phase of testing the new technology of stripping by tearing the rock near the bottom of the hole: (**a**) undercutting anchor components: 1—anchor head, 2—drive screw, 3—resilient undercutting sleeve with 6 undercutting segments; (**b**) view of the undercutting anchor head.

In the first part of the work, a modified anchor of the Hilti HDP-A type was used (Figure 14) where the modification consisted only in allowing the movement of the head (1) on the drive bolt (2) by destroying the factory-made fixed connection in the thread, fixing the head on the end of the bolt.

**Figure 15:**
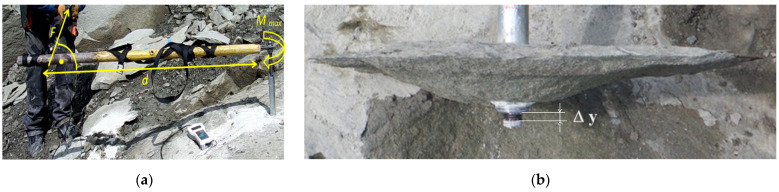
The course of field tests in the Brenna mine: (**a**) *F*—force acting on the arm *d*, necessary to rotate the anchor drive bolt, *M_max_*—torque turning the bolt; (**b**) Δy—axial displacement of the head on the drive bolt at the time of complete detachment of the rock mass in the form of the so-called cone of destruction.

During testing, the anchor embedment followed a procedure similar to that used by Hilti [26]. In the Hilti procedure, the undercutting of the rock and the expansion of the end of the sleeve on the head of the undercutting anchor occurs through simultaneous rotation and axial pressure on the expansion sleeve (3), as shown in Figure 14 (percussive-rotary process). The final result is the wedging of the anchor head in the undercut, which prevents it from being pulled out. Exceeding the load capacity of the mechanical connection formed in this way leads to either the anchor being pulled out along with the detached rock element or its breakage. In the first version of the modified Hilti anchor, the embedding and spreading of the head were carried out in a similar manner. However, after the anchor was embedded in the hole, further screwing of the propeller (2) into the head (1) (Figure 14a) led to the fracturing of the rock, caused by the formation and propagation of a crack near the largest diameter of the conical head. In the final phase, this resulted in the detachment of a lump of rock, forming the so-called cone of destruction (Figure 15b).

Studies [37,90] have also shown that turning the propeller of the modified anchor requires a significant torque (*M_max_*, Figure 15a) which, for a typical value of the force *F* produced in a human hand (200–300 N), requires a suitably long lever (*d*, Figure 15a). Such a situation would lead to the elimination of the proposed technology from use in mining rescue operations carried out in confined space conditions.

The initial experiment involving the extraction of rock fragments using a modified anchor was inspired by observations made during a study on the extraction of undercutting anchors embedded in rock using conventional methods. In the traditional approach, extracting the anchor and detaching the rock mass required the use of a large frame to support the hydraulic cylinder, which generates the force needed for anchor removal. This frame is heavy, primarily due to the wide spacing needed between the frame’s support points and the rock to minimize their impact on the stress distribution within the rock.

A crucial observation suggested that the extraction process could be performed differently by utilizing the anchor’s tearing action near the bottom of the borehole, functioning similarly to a screw mechanism. This method eliminates the need for a supporting frame or structure for the actuator, as the process is driven by applying torque to the bolt, which is then converted within the bolt mechanism into an axial force exerted on the anchor’s nut or head. As a result, the device’s design is greatly simplified, though a specific amount of torque is required. Preliminary studies aimed to estimate the magnitude of this torque for a given anchoring depth and rock strength parameters. A significant challenge lies in estimating the friction coefficient between the head’s components (such as the thread) in the presence of fragmented rock formations, as well as the friction between the rock and the conical surface of the head. Therefore, the simulation attempted to align the rock’s physico-mechanical parameters with the potential values observed in field tests.

As shown in Figure 15b, the shape of the failure surface and the crack propagation trajectory during rock ripping is similar to that observed during the pullout of the typical HDP-A anchors implemented at the Brenna mine (weak gray sandstone), e.g., [91]. This type of detachment was dominant; however, instances of changing the initiation point of crack propagation were observed (as seen in Figure 13).

**Figure 16:**
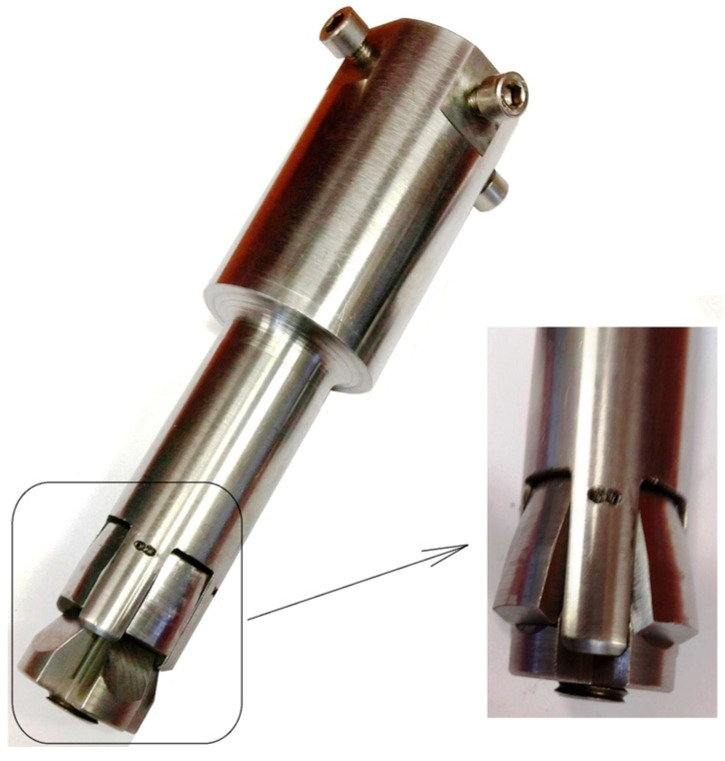
Detachment head with a drive torque multiplier.

Studies have shown that, in the case of the manual driving of anchors during their spreading, it is necessary to use a force/torque multiplier and further modify the design of the pull-off anchor, leading to the development of a completely new pull-off tool (pull-off head), as illustrated in Figure 16. The working part of the proposed head is a suitably modified version of the conical head of a classic pull-off anchor. As shown in Figure 16, the head is equipped with a torque multiplier (6) and an outwardly extending profiled drive pin (16). Unlike the Hilti anchor head, the expansion sleeve, which originally had six segments, is replaced by four conical segments that spread mechanically. After spreading in a pre-drilled hole with a conical undercut, the head adopts a conical shape, similar to the Hilti anchor design. The angle of the cone head *β* (Figure 16) after expansion is approximately 13.5°. This angle was chosen to increase the potential extent of the failure zone in the rock medium, as indicated by previous numerical studies and experimental validation [59].

**Figure 17:**
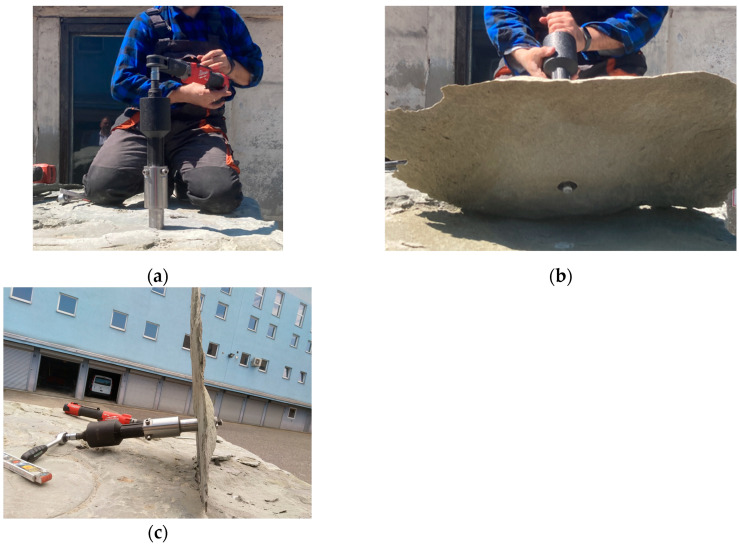
Separation tests in sandstone and an upgraded anchor with a torque multiplier: (**a**) implementation of the anchor pullout process using the designed drive system, (**b**) view of the stripped rock element, (**c**) view of the anchor drive mechanism with the disconnected rock element.

For the physical model of the head that was made, undercutting tests were carried out for holes with depths in the range of 50–100 mm (Figure 17). This corresponds to an undercut depth of 30–80 mm. The effective depth of anchoring was determined by the depth at which the onset of propagation of the de-bonding fracture occurred. In the case of the onset of propagation of the detachment fracture near the bottom of the hole, the total depth of the hole was taken as the *h_ef_*. The tests in most cases ended with a breakaway at a torque applied to the multiplier in the range of 13.5–18.0 Nm, which, after performing the relevant conversions, corresponds to an axial breakaway force of 212–283 kN. These values correspond to the ranges of forces, obtained in the baseline project, during field tests of pullout undercutting anchors in the Brenna mine.

**Figure 18:**
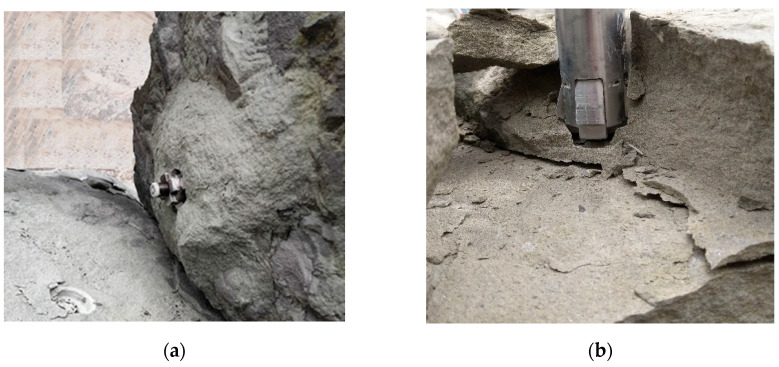
Localization of the slot initiation zone during the breaking out of the cone of damage with the use of a breakout anchor: (**a**) at the head conical base, (**b**) at the base of the anchor driving screw.

As can be seen in Figure 17, the shape of the failure zone/damage surface in this case also does not differ from that observed during the pullout of Hilti undercutting anchors at the Brenna mine (e.g., [54]) or a modified version of the stripping anchor by tearing through the rock near the bottom of the hole [50,92]. However, it was possible to register the occurrence of different areas of crack initiation during stripping, as shown in Figure 18.

In Figure 18, characteristic cases of the initiation and development of slots during the breaking out of bigger rock lumps are presented.

The tests showed that, in the predominant form of breakouts, the initiation of the slot occurred at the conical base of the head, as shown in Figure 18a. Occasionally, breakouts with the slot initiating at the base of the driving screw (as in Figure 18b) were observed. The second form was primarily associated with the bedding of grey sandstone along planes perpendicular to the anchor axis, which significantly facilitated the destruction of the structure by breaking the rock.

## 4. Discussion

The test results presented confirm the tendencies in the propagation and initiation of cracks during the failure of the rock medium, resulting from the action of the anchor head, which breaks rock fragments by fracturing the medium at the base of the driving screw of the anchor head, as observed during the tests.

The primary limitations of the model used thus far stem from the originally adopted rock failure mechanism, i.e., the cohesive zone model. This approach was a consequence of the simplifications in anchoring practices, reflecting the current state of knowledge. Our research has significantly expanded this understanding, and the insights gained now allow for a long-term shift in the modeling approach. This shift takes into account the mixed-mode crack propagation in the rock, which was observed in the field experiments.

**Figure 19:**
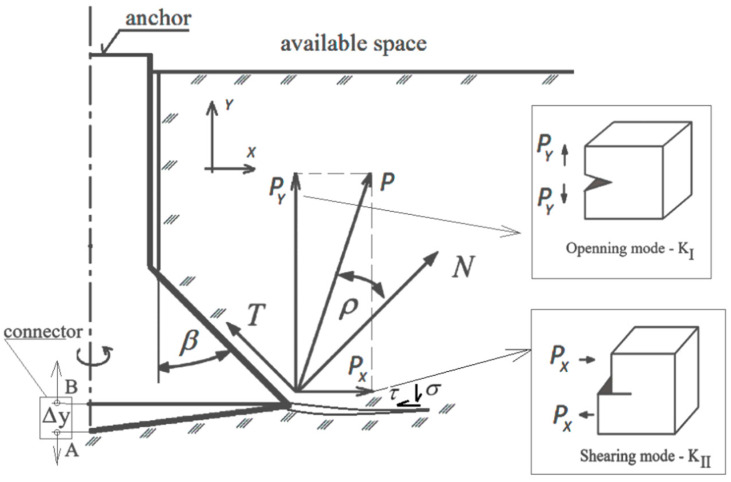
Action impact of the components *P_x_* and *P_y_* for the interaction of the anchor conical head and the rock: *β*—head angle, *ρ*—friction angle of the head and the rock; *T*—force of friction of the head against the rock (tangential component); *N*—normal component to conical undercutting of the rock; *P*—resultant force of the conical head action on the rock; *P_x_* and *P_y_*—components of the force of the anchor acting on the rock in the accepted coordinate system *XY*, where *Y* is in accordance with the anchor axis; *σ*—normal stress in the slot top; and *τ*—tangential stress, A, B—connector nodes.

As it can be concluded from Figure 19, the action strength of the conical head on the *P* anchor can be decomposed into the components *F_x_* and *F_y_* (in the accepted coordinate system). The action of the *F_y_* component causes the generation of Mode I of cracking (fracture mode—tensile opening), whereas the *F_x_* component is in favor of generating Mode II of cracking (fracture mode—in plane shear). During the development of the slot, the significance of shear increases with the result of bending the broken-out element due to an increasing slot length. The significance of tangential stresses for slot propagation during the generation of a fracture cone was discussed, among others, in the publication of [93].

As it can be concluded from tests and research on the cracking of brittle media (including sandstone), in complex cases of load and in the occurrence of complex cases of cracking [94,95,96,97,98,99] there is a significant change in the stress-intensity factor in Mode I and II (*K_IC_*, *K_IIC_*) as well as in the critical strain energy release rate (*G_IC_*, *G_IIC_*). Relative to the changes in the acting load direction, the ratio of these parameters changes. However, in the conducted analyses the impact of this aspect has not been taken into consideration.

## 5. Conclusions

All tested anchors were characterized by a conical head, the geometry of which is responsible for forming the contact zone with the rock and determining how loads are transferred to the rock. It was found that the various anchor head designs used (with a typical six-element undercutting sleeve or a four-element sleeve used in the final design solution), with a standard head angle value *β*, resulted in a comparable formation and extent of the failure zone in the upper region. Differences were observed only in the zone beneath the head, where the end of the propeller is potentially affected, introducing additional stress concentrations below the anchor.

The relationship between the propagation of cracks, the coefficient of friction between the anchor head and the rock, and the effect of fracture energy on the propagation of cracks accompanying the fracture of the rock medium was confirmed. A higher coefficient of friction between the anchor head and the rock limits the extent of the damage zone and causes the damage to reach the rock’s free surface more quickly. The effect of fracture energy on the extent of the fractures is not uniform. A reduction in fracture energy, while maintaining tensile strength, leads to more brittle material behavior, which in turn reduces the extent of the cracks. Additionally, further clarification is needed regarding the initiation points of cracks in the case of breakout anchors and rock failure at the base of the anchor driving screw. This aspect should be considered, keeping in mind the possibility of bimodal cracking of the rock as a result of the action of the anchor’s conical head.

## Data Availability

Data presented in this study is available from corresponding authors upon request.
